# Disseminated Exfoliative Dermatitis Associated with All-Transretinoic Acid in the Treatment of Acute Promyelocytic Leukemia

**DOI:** 10.1155/2012/236174

**Published:** 2012-07-17

**Authors:** Yonal Ipek, Dogru Hulya, Aktan Melih

**Affiliations:** Division of Hematology, Department of Internal Medicine, Istanbul Medical Faculty, Istanbul University, 34093 Istanbul, Turkey

## Abstract

Acute promyelocytic leukemia (APL) is a biologically and clinically separate type of acute myeloid leukemia characterized by a translocation involving the retinoic acid receptor-alpha (RARa) locus on chromosome 17, the great majority of which is t(15; 17)(q24.1; q21.1) (Collins (1998), Melnick and Licht (1999), and Grimwade (1999)). Retinoic acid is a critical ligand in the differentiation pathway of multiple tissues, mediated through binding to an RAR. All-trans retinoic acid (ATRA) is a subgroup of the retinoid family, which induces complete remission (CR) in APL by causing differentiation and apoptosis in immature malignant promyelocytes rather than inducing cell death by cytotoxicity (Warrell et al. (1993), Liu et al. (2000), and Cassinat et al. (2001)). ATRA-associated toxicity consisting of headache, fever, weakness, fatigue, dry skin, dermatitis, gastrointestinal disorders, and hypertriglyceridemia has been shown to be mild (Kurzrock et al. (1993)). Herein, we describe a patient with APL that developed an erythematous reaction of the whole body followed by desquamation and exfoliation during ATRA therapy.

A 57-year-old woman presented one year ago with ecchymoses scattered over the entire body. Physical examination revealed pallor and multiple ecchymoses on her arms, legs, and trunk. A full blood count showed a haemoglobin concentration (Hb) of 11 g/dL, white blood cell (WBC) count of 6450/mm^3^, and a platelet count of 30000/mm^3^. The laboratory results were as follows: LDH 3763 U/L (normal range, 240–480), D-dimer 2679 ug/L (normal range < 500 ug/L), and fibrinogen 93 mg/dL (normal range, 180–350 mg/dL). On peripheral blood smear, malignant promyelocytes comprised 60% of the cells (Figure 1) and the bone marrow aspiration yielded a hypercellular marrow with increased malignant promyelocytes. Immunophenotypic analysis of bone marrow was positive for CD13, CD33, and CD45 and negative for HLA-DR. Cytogenetic analysis showed a *t*(15,17) (q22; q21) karyotype in all metaphases examined. The final diagnosis was intermediate-risk acute promyelocytic leukemia. The patient was started on induction therapy with oral all-trans retinoic acid (ATRA) 45 mg/m² per day until complete remission (CR) and intravenous idarubicin 12 mg/m² on days 2, 4, 6, and 8 (PETHEMA LPA99 trial) [1]. Disseminated intravascular coagulation was treated with fresh frozen plasma (FFP). Over the next ten days, fibrinogen levels returned to normal and FFP was stopped. On day 28, there were no myeloblasts on peripheral blood smear and bone marrow examination and cytogenetic analysis showed a normal karyotype. Seven days after CR, first consolidation course with idarubicin (7 mg/m² per day for 4 days) combined with ATRA (45 mg/m² per day for 15 days) was initiated. Six days after completion of consolidation therapy, xerosis ensued. A few days later, a generalized erythema over the entire body appeared, which over a 24-h period rapidly transformed to dry desquamation and exfoliation predominantly on the palmar and plantar areas (Figures 2, 3, and 4). Other toxicities attributable to ATRA were increased cholestatic enzymes with alkaline phosphatase 418 IU/L (normal range, 30–135 IU/L) and gamma-glutamyl transferase 727 IU/L (normal range, 5–85 IU/L) and depression. The drug was halted due to severe dermatologic, hepatic toxicity and depression. Within two weeks, liver enzymes returned to normal and by the third week, the skin toxicity remarkably improved after using topical emollients. The second and third consolidation courses consisted of mitoxantrone monotherapy (10 mg/m² per day for 5 days) and idarubicin monotherapy (12 mg/m² per day for 2 consecutive days), respectively. The patient is still in CR and under followup in our hematology department on maintenance therapy with oral mercaptopurine (50 mg/m² per day), oral methotrexate (15 mg/m² per week), and oral ATRA (45 mg/m² per day for 15 days every three months). 

Retinoids, the natural and synthetic derivatives of vitamin A, exert their effects by modulating the transcription of genes critical to cellular differentiation by binding to nuclear RAR [2]. ATRA acts on RAR and induces differentiation and apoptosis in both normal and malignant promyelocytes [3, 4]. Side effects of ATRA are dose related and mostly involve the skin and mucous membranes. Dermatologic complications consist of skin/mucous membrane dryness (77%), rash (54%), pruritus (20%), alopecia (14%), and skin changes (14%). Most patients complain of dryness and at higher doses, erythema of the skin, mouth, eyes, and lips. A few cases of scrotum exfoliative dermatitis with ulcers associated with ATRA treatment have been described [2, 5, 6]. In most of these cases, the toxicities were noted in patients at dose levels around 150 mg/m² per day [2]. On the contrary, our patient developed disseminated exfoliation and desquamation at a dose of 45 mg/m² per day. Exfoliative dermatitis, a rare and potentially serious skin disorder, refers to the skin that is diffusely red and inflamed with varying degrees and types of scaling. Exacerbation of an underlying skin disease, drug reactions, and underlying malignancies are the most common causes. In our patient, the development of exfoliative dermatitis was attributed to ATRA and three weeks after discontinuation of the drug, the skin toxicity significantly improved, in accordance with observations in previous reports [2]. Skin toxicity is an encountered side effect of ATRA treatment. Yet, to the best of our knowledge, such a severe dermatologic adverse effect attributed to ATRA has not been reported before. Previous data had reported that the side effects of ATRA, other than ATRA syndrome, were dose related [2]. Our case, in contrast, demonstrates that severe dermatological toxicity can develop at lower doses as well. Muindi et al. reported that the plasma concentrations of ATRA decrease significantly after 2 to 6 weeks of continuous therapy [7]. On the contrary in our case, no adverse effects were encountered during the 28-day induction therapy with ATRA. Yet, a severe dermatologic complication developed after the first consolidation therapy on day 56. Thereupon, ATRA was not included in the following consolidation therapies. Under close followup, oral ATRA was added to the maintenance therapy and skin toxicity did not recur. Our observations demonstrate that the exact pathogenesis of skin toxicity due to ATRA still remains unclear and likely involves a complex interaction of many factors including immunology, genetics, and drug metabolism.

## Figures and Tables

**Figure 1:**
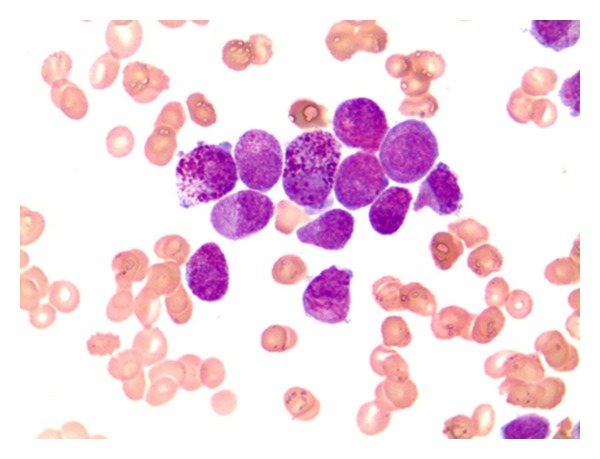
Heavily granulated promyelocytes and auer rods in peripheral blood.

**Figure 2:**
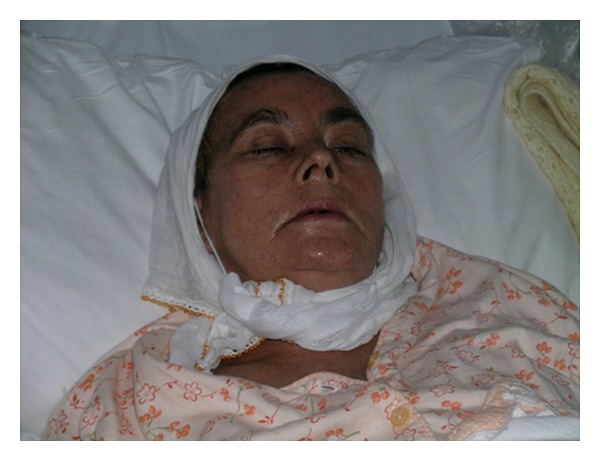
Widespread facial erythema.

**Figure 3:**
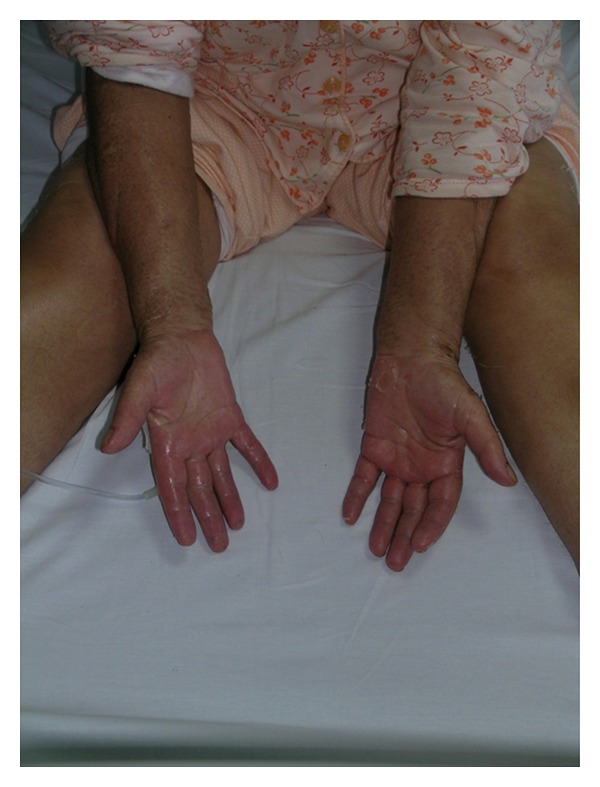
Erythema of the palms with generalized exfoliative desquamation.

**Figure 4:**
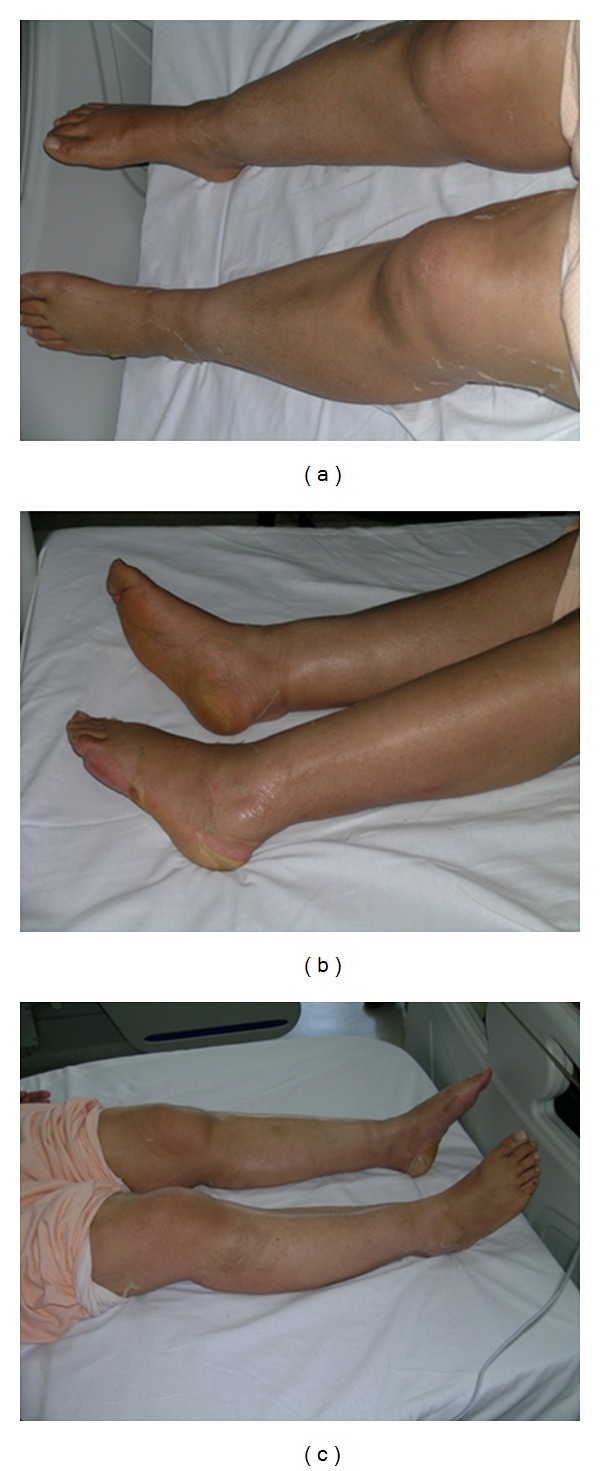
Diffuse erythema and desquamation most evident at the plantar surfaces of the lower extremities.

## References

[1] Sanz MA, Montesinos P, Vellenga E (2008). Risk-adapted treatment of acute promyelocytic leukemia with all-trans retinoic acid and anthracycline monochemotherapy: long-term outcome of the LPA 99 multicenter study by the PETHEMA Group. *Blood*.

[2] Kurzrock R, Estey E, Talpaz M (1993). All-trans retinoic acid: tolerance and biologic effects in myelodysplastic syndrome. *Journal of Clinical Oncology*.

[3] Gringnant FR, Ferruchi PF (1993). The acute promyelocytic leukemia specific PML/RAR fusion protein inhibits differentiation and promotes survival of myeloid precursor cells. *Cell*.

[4] Metcalf D (1989). The molecular control of cell division, differentiation commitment and maturation in haemopoietic cells. *Nature*.

[5] Ketsueki R (1998). Scrotum exfoliative dermatitis with ulcers associated with treatment of acute promyelocytic leukemia with all-trans retinoic acid. *The Japanese Journal of Clinical Hematology*.

[6] Guan-Lin S (1993). Treatment of acute promyelocytic leukemia (APL) with all-trans retinoic acid (ATRA): a report of five-year experience. *Zhonghua Zhong Liu Za Zhi*.

[7] Muindi J, Frankel SR, Miller WH (1992). Continuous treatment with all-trans retinoic acid causes a progressive reduction in plasma drug concentrations: implications for relapse and retinoid “resistance” in patients with acute promyelocytic leukemia. *Blood*.

